# Experimental Cell Models for Investigating Neurodegenerative Diseases

**DOI:** 10.3390/ijms25179747

**Published:** 2024-09-09

**Authors:** Cecilia Evangelisti, Sherin Ramadan, Antonio Orlacchio, Emanuele Panza

**Affiliations:** 1 Medical Genetics Unit, IRCCS Azienda Ospedaliero-Universitaria di Bologna, 40138 Bologna, Italy; cecilia_evangelisti@aosp.bo.it; 2 Department of Medical and Surgical Sciences, University of Bologna, 40138 Bologna, Italy; sherin.ramadan@studio.unibo.it; 3 Department of Medicine and Surgery, University of Perugia, 06123 Perugia, Italy; antonio.orlacchio@unipg.it; 4 Laboratory of Neurogenetics, European Center for Brain Research (CERC), IRCCS Santa Lucia Foundation, 00143 Rome, Italy

**Keywords:** primary cells, iPSCs, model organisms, organoid, gene editing

## Abstract

Experimental models play a pivotal role in biomedical research, facilitating the understanding of disease mechanisms and the development of novel therapeutics. This is particularly true for neurodegenerative diseases, such as Alzheimer’s disease, Parkinson’s disease, Huntington’s disease, amyotrophic lateral sclerosis, and motor neuron disease, which present complex challenges for research and therapy development. In this work, we review the recent literature about experimental models and motor neuron disease. We identified three main categories of models that are highly studied by scientists. In fact, experimental models for investigating these diseases encompass a variety of approaches, including modeling the patient’s cell culture, patient-derived induced pluripotent stem cells, and organoids. Each model offers unique advantages and limitations, providing researchers with a range of tools to address complex biological questions. Here, we discuss the characteristics, applications, and recent advancements in terms of each model system, highlighting their contributions to advancing biomedical knowledge and translational research.

## 1. Introduction

Biomedical research relies heavily on experimental models to recapitulate disease phenotypes to elucidate the underlying mechanisms of disease and to develop effective therapeutic interventions. Over the years, scientific research has developed a multitude of model systems to mimic human physiology and pathology. These range from simple patient cell lines to unicellular organisms, like yeast, and complex 3D organoid models and multicellular organisms, such as zebrafish or mice. Primary cells from patients represent a great model for many diseases, but neurodegenerative conditions are particularly demanding in terms of modeling. In fact, while nervous system cells cannot be obtained directly from patients, in vitro modeling (for instance, with neuron models derived from induced pluripotent stem cells) can be particularly expensive. Motor neuron diseases (MNDs), and more generally neurological disorders (NDs), represent a heterogeneous group of disorders characterized by the progressive degeneration of neurons in the brain and spinal cord. These conditions encompass a spectrum of diseases, including, Parkinson’s disease (PD), Alzheimer’s disease (AD), amyotrophic lateral sclerosis (ALS), hereditary spastic paraplegia (HSP), and Huntington’s disease (HD).

Parkinson’s Disease (PD). PD is one of the most common neurodegenerative disorders and it is characterized by the progressive loss of dopaminergic neurons in the substantia nigra region in the midbrain, thus leading to tremors, impairment of fine motor coordination, muscle rigidity, and cognitive decline. From a histopathological point of view, the presence of Lewy bodies, constituted by aggregates where α-synuclein is a major component, in dopaminergic neurons is considered the major hallmark of PD [1,2]. Most PD cases are sporadic, while inherited forms account for the remaining 5–10% of cases. Several genes are known to be involved in PD etiopathogenesis, namely *SCNA*, *LRRK2*, *PINK1*, *PARK2*, *GBA1*, *DNAJC6*, cytoplasmic protein sorting 35 (VPS35), and *DJ-1* [3,4,5].

Alzheimer’s Disease (AD). AD is the most frequent cause of dementia and it has been estimated to affect 50–55 million people worldwide. In AD, a severe loss of neurons in the cerebral cortex occurs and this, in turn, impairs cortical function. As for the other neurodegenerative diseases, familial forms (FAD) account for only a minority of cases and the most common mutations are found in presenilin 1 (PSEN1), presenilin 2 (PSEN2), and the amyloid precursor protein (APP). Genome-wide association studies have uncovered several risk factors, with allele 4 of apolipoprotein E (APOE4) showing the strongest association with sporadic, late-onset AD [6]. AD patients exhibit deposition of β-amyloid plaques and neurofibrillary tangles in the brain tissue, as well as hyperphosphorylation and aggregation of the Tau protein, which is the main actor in disease progression. These two cellular phenomena lead to structural changes in the brain, resulting in neuronal destruction and synaptic impairment. Since the accumulation of β-amyloid precedes the accumulation of tau pathology and tau-mediated neurodegeneration in AD, several therapeutical options focusing on the removal of Aβ from the brain have been developed. However, Aβ-reducing therapies alone are not able to hinder the progression of tau-mediated neurodegeneration in patients with AD [7], underlining that different therapeutical approaches are needed and that many aspects of AD etiopathogenesis are still obscure.

Amyotrophic Lateral Sclerosis (ALS). ALS is a late-onset, chronic, and progressive disorder that causes degeneration of upper and lower motor neurons (MNs), thus resulting in paralysis, respiratory failure, and death [8]. Mutations in more than 30 different genes, including superoxide dismutase 1 (*SOD1*), TAR DNA-binding protein 43 (*TARDBP*), fused in sarcoma (*FUS*), and the intronic hexanucleotide repeat expansion in the *C9orf72* gene, are currently associated with both familial and sporadic ALS, even though sporadic cases account for 90–95% of all cases [8]. In addition, other genes related to ALS were found to be related to the autophagic system, including *P62*, *OPTN*, *VCP*, *UBQLN2*, and *TBK*.

Hereditary Spastic Paraplegia (HSP). HSP is a group of inherited neurodegenerative disorders characterized by progressive spasticity and weakness of the lower limbs. Although the degeneration of motor neurons in the pyramidal tract is the main characteristic of HSP, a thin corpus callosum and intellectual disability are also reported in patients [9]. Nowadays, more than 87 forms have been identified, caused by mutations in at least 73 different genes. Autosomal dominant, autosomal recessive, X-linked, and mitochondrial inheritance forms have been reported [10].

Huntington’s Disease (HD). HD is a neurodegenerative, autosomal dominant disorder that is characterized by involuntary choreatic movements, with cognitive and behavioral disturbances. HD is caused by an expansion of CAG repeats in the first exon of the huntingtin (*HTT*) gene. In normal individuals, the range of repeat numbers is 9 to 36, while affected individuals show more than 37 repeats, leading to a HTT protein with a long polyglutamine expansion that is more prone to aggregate and it is believed to induce neurodegeneration through abnormal interactions with other proteins, leading to many cellular alterations and ultimately cell death [11]. Despite advances in our understanding of MND pathophysiology, effective treatments remain elusive, highlighting the urgent need for innovative research approaches. Experimental models play a pivotal role in deciphering the underlying mechanisms of MNDs, from elucidating disease etiology to identifying potential therapeutic targets. In this article, we provide an overview of the experimental models commonly employed in MND research, highlighting their strengths, limitations, and contributions to advancing our understanding of these devastating disorders. Here, we focused on three major categories of experimental cell models: primary patient cell lines, induced pluripotent stem cells, and organoids. Each model system offers distinct advantages and applications, contributing to our understanding of various diseases and facilitating drug discovery and development.

## 2. Main Text

### 2.1. Primary Patient Cell Lines

Primary patient cell lines, derived directly from patient samples, as fibroblasts, represent an essential tool for studying disease mechanisms and drug responses in a relevant biological context. These cell lines retain many of the genetic and phenotypic characteristics of the original tissue, making them ideal for personalized medicine approaches.

**Figure 1:**
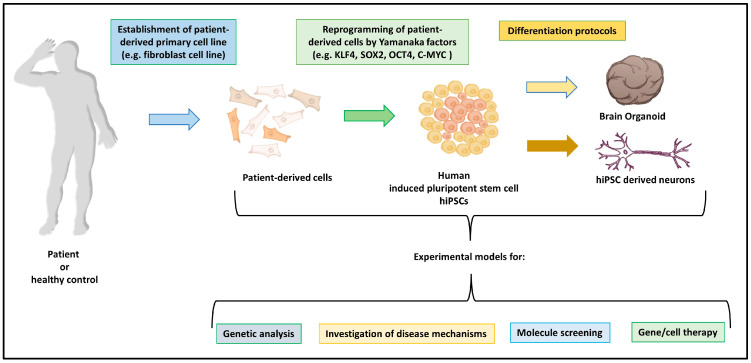
Schematic representation of the process to generate experimental models.

Are patient’s skin fibroblasts a good model for investigating MNDs? The straightforward answer to this question is very simple and it can be reformulated with another question: is your gene of interest expressed in the primary cell you are working on? (Figure 1). This question can be answered if we are working with conditions where the genetic cause is known. In a study, the value of these cells has been quantitated by analyzing and comparing the gene expression of causative genes for MNDs in fibroblasts to lymphocytes transformed by the EBV virus (Ebstein Barr virus), cortical neurons, and iPSCs [12]. In general, the data show that distinct genes can have a different expression in different cell types. While some MND genes are highly expressed in fibroblasts [13,14], others have a higher level of expression in cortical neurons, indicating that, in general, a preselection of the proper cellular model is necessary to potentially decipher the molecular mechanisms with pathophysiological relevance and to screen for novel therapeutic targets in various types of MNDs [15].

Nevertheless, patient-derived fibroblasts have been widely utilized as valuable models for studying various conditions, and the use of skin fibroblasts represents an exceptional tool to investigate neurodegenerative processes [14,16].

Skin fibroblasts are peripheral cells that can be accessed through a patient biopsy, using a simple procedure, with a relatively low level of discomfort for the individual. These biopsies are then cultivated in vitro to expand the fibroblast component, a procedure that can take roughly four weeks [17].

While a more complex role of fibroblasts in the central nervous system has been suggested recently [18], these cells have a clear connective tissue origin. Despite this fact, these cells maintain metabolic and biochemical relationships with neurons. These cells have been used either to study early alterations of specific organelles, as the mitochondria [19], or in regard to the general phenotype of different neurodegenerative conditions, such as AD, PD, HD, and ALS [20,21,22,23].

Fibroblasts derived from PD patients with mutations in genes, such as *PINK1*, *Parkin*, and *DJ-1*, have been employed to investigate mitochondrial dysfunction, a key pathological feature of PD. These studies have revealed abnormalities in mitochondrial morphology, function, and dynamics, providing insights into disease mechanisms and potential therapeutic targets [24]. Similarly, fibroblasts from patients with familial AD forms carrying mutations in the amyloid precursor protein (APP) showed significantly higher levels of heat-shock proteins, HSP90 and HSP70, as confirmed by Western blot analysis [25]. In the same study, it was shown that fibroblasts from patients with familial AD forms, with mutations in the *Presenilin 1* gene, exhibited changes in signaling pathways associated with cellular stress, autophagy, lysosomes impairment, and tau phosphorylation. Therefore, these observations indicate that fibroblasts could be a valuable tool in modeling neurodegeneration-related pathways and identifying early biomarkers for Alzheimer’s disease [25]. As an example, fibroblasts from patients with tauopathies, such as frontotemporal dementia (FTD) and progressive supranuclear palsy (PSP), have been employed to study tau protein aggregation and toxicity. For instance, the findings of a study by Ibanez-Salazar and colleagues [26] suggested that the phosphorylation state and accumulation levels in subcellular compartments of Tau are modified by oxidative stress through mechanisms, which despite not being completely understood, have been observed previously in neuronal models of tauopathies [27].

In another study, fibroblasts derived from HD patients with naturally occurring, expanded CAG repeats in the huntingtin gene were used to investigate classic HD phenotypes. These phenotypes included altered morphology, size, growth rate, increased sensitivity to oxidative stress, abnormal adenosine diphosphate/adenosine triphosphate (ADP/ATP) ratios, and hypo-phosphorylated huntingtin protein. Additionally, dysregulated reactive oxygen species (ROS)-dependent huntingtin localization to nuclear speckles was observed in HD cells. This study demonstrates the generation and characterization of a human, clinically relevant cellular model for investigating disease mechanisms in HD at the single-cell level, which preserves critical functions for huntingtin transcriptional regulation and genomic integrity, unlike transformed cell lines [28].

Also, in the case of ALS, fibroblasts have been derived to better study this condition. It is known that, during the presymptomatic stage, perivascular fibroblast cells show the strongest gene enrichments and their marker proteins, SPP1 and COL6A1, accumulate in enlarged perivascular spaces in patients with sporadic ALS, suggesting that the activity of perivascular fibroblasts can predict the survival of ALS patients [29]. Of note, fibroblasts from ALS patients with mutations in *SOD1*, *TARDBP*, and *FUS* genes have been used to investigate motor neuron vulnerability to cellular stressors.

In a study, the primary hallmark of many forms of familial and sporadic ALS was investigated, which concerns the reduction in the nuclear TDP-43 protein and its inclusion in cytoplasmic aggregates in motor neurons. To understand the cellular and molecular mechanisms underlying TDP-43 mislocalization, human skin fibroblasts were examined from individuals with familial ALS, both patients with TDP-43 mutations and individuals with sporadic ALS but without TDP-43 mutations or mutations in other ALS-related genes. It was found that all ALS fibroblasts exhibited partial cytoplasmic localization of the TDP-43 protein and reduced cell metabolism compared to fibroblasts from apparently healthy individuals. Furthermore, ALS fibroblasts showed reduced stress granule formation in response to H_2_O_2_ stress. These findings reveal specific cellular and molecular defects, providing insight into the mechanisms involved in degenerating motor neurons [30].

A recent article shows the importance of fibroblast cell lines, which in combination with other in vitro and in vivo models, helps to elucidate the pathogenetic mechanism of a novel form of HSP [31]. In fact, patient fibroblasts bearing *AMFR* homozygous mutations were used to demonstrate an alteration in lipid and cholesterol metabolism and impairment of the endoplasmic reticulum (ER) membrane. The use of this cell model helped to identify measurable phenotypes, as the cell size (the median lipid droplet size in patient-derived cells was significantly larger compared to wild-type controls) is useful to investigate treatments to ameliorate the disease phenotype [31]. Interestingly, it has been observed that treatment with FDA-approved HMG-CoA reductase inhibitors, simvastatin and atorvastatin, can rescue the phenotypes seen in *Amfra−/−* zebrafish. This result suggests a potential pathway for precision medicine in treating this newly identified disorder [31].

Patient-derived fibroblasts were employed as screening platforms to identify potential therapeutic compounds, in the case of another neurodegenerative condition, multiple sulfatase deficiency (MSD, OMIM 272200) [32]. An FDA-approved drugs screening, using immortalized MSD patient fibroblasts, demonstrated that the use of retinoids (tazarotene and bexarotene) led to the correction of MSD pathophysiology [33].

**Figure 2:**
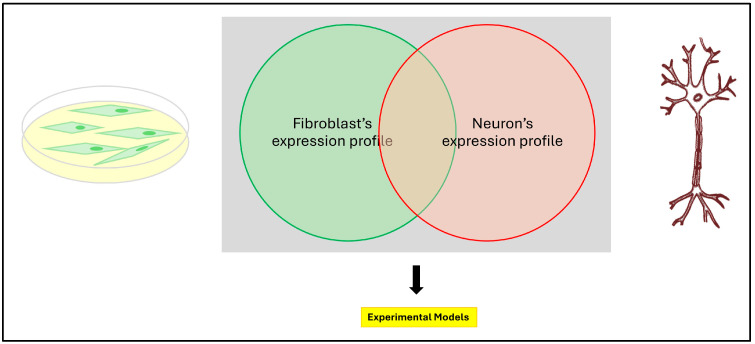
Primary cell models for investigating neurodegenerative conditions share genes of interest expression profile.

Overall, patient-derived fibroblasts serve as valuable tools for modeling neurodegenerative diseases, recapitulating key aspects of disease pathology and providing platforms for mechanistic studies and drug discovery efforts (Figure 2).

### 2.2. Human Induced Pluripotent Stem Cells (iPSCs)

Induced pluripotent stem cells (iPSCs) represent a promising alternative to the use of embryonic stem cells (ESCs), which until recently were the main source of pluripotent stem cells. The distinctive quality of pluripotent cells is their ability to self-renew and proliferate into all adult cell types during the differentiation process.

IPSCs can be obtained from different somatic cells (fibroblasts, lymphocytes, urinary tract-derived cells, for instance) and this is one of the greatest advantages of this kind of pluripotent stem cell. The use of somatic cells makes the generation of pluripotent stem cells more accessible, by bypassing ethical issues concerning embryo-derived tissues. Moreover, these cells can be directly derived from the donor in the examination/recipient of the therapy. Deriving these cells directly from the recipient makes disease modeling both more reliable and, in the case of cell-based therapies, highly compatible with the same recipient, by reducing the risks associated with allogenic stem cell transplants [34]. The genes and factors that are used to reprogram somatic differentiated cells into iPSCs include Yamanaka factors. Some of the available protocols include the use of *SOX2*, *OCT4*, *NANOG*, and *LIN28* genes to generate human iPSCs [35], in others, the combination of *KLF4*, *SOX2*, *OCT4*, and *C-MYC*, has been used [36]. The delivery of these factors can employ viruses, such as the MMLV or SENDAI virus, but also liposomes and piggyBac transposons [37].

One promising application of iPSCs consists of modeling diseases. Furthermore, the differentiation of neural cells from iPSCs could help to elucidate the complex mechanisms that lead to their development during cell differentiation. Cell lines derived from these iPSCs would not only be disease specific, but also patient specific, bearing different pathogenetic variants. Ultimately, once a disease-representing iPSC line is established, these lines could serve as models for targeted drug therapies. Finally, iPSCs can serve as a source of cell-transplantation treatments entirely derived from patients. This could help in regenerating damaged tissues and reversing or ameliorating the course of disease [38].

Moreover, iPSCs can be directed towards differentiation in neural stem cells (NSCs), by finely tuning molecular mechanisms by means of specific growth factors and/or the inhibition and stimulation of several signaling pathways. For example, among these, there are bone morphogenetic protein-4 (BMP-4), transforming growth factor-beta (TGFβ), and Sma-and Mad-related proteins (SMAD). Additionally, iPSC-derived NSCs can be further differentiated into various neuron types, each characterized by distinct functions. So far, multiple neuronal cell types have been successfully differentiated: cortical neurons, dopaminergic neurons, oligodendrocytes, astrocytes, motor neurons, hippocampal neuros, and serotoninergic neurons. In addition to cells of neuronal origin, iPSCs can also be directed to obtain microglia, monocytic lineage-derived cells that represent the innate immune system of the central nervous system that play crucial roles in neurodegenerative diseases. For this reason, specific differentiation protocols towards this type of cell have been developed [39], with protocols including specific factors, for example CSF-1, IL-34, and TGFb administration [40].

Using human induced pluripotent stem cells to model neurodegenerative diseases also solves the problem of needing to have the brain tissue of a patient at your disposal. The contribution of disease modeling based on iPSCs is particularly relevant also in the context of rare diseases and, particularly, in neurodegenerative diseases. Although most neurodegenerative diseases are sporadic, hereditary forms have been described as well. A distinct aspect of inherited forms is that the clinical symptoms and neuropathological findings are often indistinguishable from those of sporadic cases, suggesting common genomic signatures and pathophysiologic mechanisms that might underlie both forms. Unfortunately, to date, very few effective therapies for these diseases are available and most of the available treatments focus on managing symptoms. The development of patient-derived iPSCs, from sporadic and familial patients, could represent a valuable contribution toward personalized therapy based on specific gene alterations [41].

In the case of AD, multiple iPSC lines have been developed to recreate the pathological mechanisms that lead to disease development and progression.

Human iPS cell-derived neuronal models have been mainly used to investigate tau propagation, as these cell lines provide insight into the pathological mechanisms of other tauopathies, as such frontotemporal dementia and progressive supranuclear palsy. Moreover, iPSC lines have been derived from FAD patients with *PSEN1* (p.Ala246Glu) and *PSEN2* (p.Asn141Leu) mutations. Neurons differentiated from this iPSC cell line initially showed an increase in β-amyloid levels; however, they lacked tau protein accumulation. Furthermore, these neurons exhibited an increase in the Aβ 42/40 ratio, mitophagy impairment, and dysfunctional mitochondria [42,43]. These results have been confirmed by other works, confirming the validity of such a model [44].

Retinoic acid (RA) was also used to differentiate iPSCs bearing the p.Ala246Glu mutation in *PSEN1*, obtained with an equivalent approach. The resulting neurons displayed an increased Aβ 42/40 ratio, along with p-tau and premature differentiation, which is one of the early pathological features of AD [45].

Another interesting use of iPSCs to study Alzheimer’s disease was recently described by Budny and colleagues. In this study, they explored the distinct effects of APOE isoforms (*APOE2*, *APOE3*, *APOE4*) on neuronal energy metabolism, using human neurons derived from APOE-isogenic iPSCs. *APOE4*, which is a major genetic risk factor for AD, was found to enhance mitochondrial ATP production in neurons, but not in the corresponding iPSCs. Interestingly, this enhancement did not correlate with changes in mitochondrial fission/fusion proteins or *APOE* levels, suggesting a unique, gain-of-function mechanism specific to *APOE4* in neurons. This application of disease modeling by iPSCs showed how different *APOE* isoforms regulate oxidative energy metabolism in a genotype-dependent and neuron-specific manner, with important implications for understanding this condition [46].

Another application of iPSCs, makes us understand how these cells can help in overcoming the difficulty in finding drugs that are effective in human trials. An article by Wang and colleagues shows a practical application of how iPSCs derived from human somatic cells with AD-linked pathogenetic or neutral variants, together with the application of gene-editing technology, are promising in vitro models for studying disease pathogenesis in relevant cell types, including human neurons. Often, promising molecules tested on animal models fail to replicate their efficacy in patients. In this context, the use of human iPSCs can represent a platform to efficiently test drugs. In the article, the authors investigated the effects of the *APOE4* variant, being, as shown before, a major genetic risk factor for AD, using human neurons derived from iPSCs. Neurons expressing *APOE4* showed higher levels of tau phosphorylation and GABAergic neuron degeneration, independent of increased amyloid-β (Aβ) production. Interestingly, *APOE4* elevated Aβ production in human neurons, but not in mouse neurons, suggested a biological difference between species and pinpointed a shortcoming of animal models in this specific application. Converting human *APOE4* to *APOE3* through gene editing reversed these harmful effects, indicating the specific toxicity of *APOE4*. Neurons lacking *APOE* behaved similarly to those with *APOE3*, and introducing *APOE4* again induced pathological traits, suggesting a gain in the toxic function of *APOE4*. This observation was specific since the reintroduction of *APOE3* did not induce the pathological phenotype. Treatment with a small-molecule structure corrector (coded as PH002) alleviated the detrimental phenotypes, suggesting that correcting *APOE4*’s pathogenic conformation could be a therapeutic strategy for AD [47]. This is an interesting application of iPSCs, serving as platform for drug discovery based on human-specific samples.

Experimental models, based on reprograming the patient’s cells into iPSCs and differentiation in neuronal cells, have also been intensively studied for Parkinson’s disease.

Indeed, several works have focused on characterizing pathogenetic variants in the α-synuclein. Functional midbrain dopaminergic neurons were generated through differentiation of iPSCs derived from a patient with a triplication in the *SNCA* gene. The PD line exhibited several disease phenotypes, including accumulation of α-synuclein, overexpression of oxidative stress markers, and sensitivity to peroxide-induced oxidative stress [48,49].

In another study, the authors investigated the role of the *VPS35* p.Asp620Asn mutation, linked to a familial form of Parkinson’s disease, termed PARK17 (OMIM 614203) [50]. The findings reveal that dopaminergic (DA) neurons, bearing the pathogenetic variants, undergo extensive apoptotic cell death. The study also shows that endosomal trafficking is nonfunctional, with slower movement and reduced fission/fusion frequencies of Rab5a- or Rab7a-positive endosomes. Additionally, retromer-transported vesicles were abnormally localized in glial cells and α-synuclein accumulation was observed in DA neurons. Again, in this study, the use of reprogrammed cells and differentiated neurons was demonstrated to be a fundamental tool in such research, which highlighted the discovery of endosomal dysfunction, cell death, and α-synuclein buildup, as key features of PD pathogenesis in PARK17 [51].

Recently, there has been an increase in data showing an important role not only of dopaminergic neurons from the substantia nigra, but also of astrocytes, in the pathogenesis of PD. A recent study shows that it is possible to generate astrocytes from iPSCs to be used as a model for studying PD pathogenesis. Two iPSC lines were generated from a patient bearing a heterozygous *GBA1* pathogenetic variant (p.Asn370Ser). These lines were created from peripheral blood mononuclear cells, using non-integrating episomal vectors. While the role of astrocytes in PD represents an open question, the generation of the patient’s specific astrocyte lines provide a valuable cell model to study the role and interactions of astrocytes in *GBA1*-associated PD [52].

As a common denominator for this group of neurodegenerative diseases, also for HD, there is the need for valuable experimental models to study disease pathogenesis and therapeutic interventions. More importantly, reliable models are needed, the results of which can be extended among different models and to patients.

In fact, a key aspect of iPSC use is the reproducibility of the results. Nowadays, many labs generate iPSCs using the reprogramming factors delivered by vectors, based on lentiviruses or Sendai viruses. One common question for experts in the field related to how similar cells are obtained with different methods. This is a legitimate question. A recent work addresses this issue by comparing the abnormalities recorded in store-operated calcium entry in HD disease-specific iPSCs-based GABAergic medium spiny neurons, a special type of inhibitory GABAergic neuron. The data collected demonstrated that there were no significant differences in the calcium influx through the store-operated channels, nor in the levels of proteins activating this type of calcium entry in neurons differentiated from iPSCs generated with lentivirus and Sendai virus approaches. This kind of study is of pivotal importance in order to combine the functional data on iPSCs for HD, but for iPSCs in general, allowing the collection of these models in biobanks that are accessible to researchers is an important tool for such research [53].

In ALS, the complex genetic bases of the disease have resulted in difficulties in creating iPSC-derived models. To solve this issue, a collaborative group has generated over 1000 iPSC lines from both healthy controls and ALS patients, accompanied by clinical and whole-genome sequencing data, marking the largest set of iPSCs differentiated into motor neurons to date. The results of this study showed how elements, such as cell composition and sex, significantly contribute to disease variability and such elements must be taken into consideration in future studies. The work represents an important group of data, valuable for the scientific community, facilitating ALS disease modeling, with extensive omics data available for these samples [54].

Despite the limits that iPSCs can present, the collective efforts to reproduce reliable cell models using these cells are a key aspect of overcoming the models’ difficulties. One problem to address is the standardization of protocols. To obtain differentiated cells, several differentiation factors could be employed for the same line; making these protocols replicable is key to generating reliable data [55]. This issue is being addressed by the creation of multiple biobanks. In these databases, cell lines obtained through reprogramming and further differentiation are being registered, detailed, and made available to the research community [56]. Often these cell lines are obtained from patient-derived cells and the clinical data related to the donors are scarce. By standardizing the protocols and adding valuable information in a database, the results obtained in different settings would be comparable and more reliable.

Another issue concerns the loss of epigenetic age due to the reprogramming itself, which might affect the obtained results, because there is a lack of background information related to aging. In this case, there is a collective struggle to replicate this feature. Losing the epigenetic age could result in a lack of correlation between the phenotype and genotype from lines obtained by donor cells. Many neurodegenerative diseases in fact have an adult onset and patient-derived cells might have features that are not possible to replicate in a reprogrammed cell. However, due to the same peculiarity, the young epigenetic age in reprogrammed cells could contribute to the discovery of early phenotypes correlated to different diseases [57].

### 2.3. Organoids

Organoids are self-organizing three-dimensional (3D) structures derived from stem cells or organ progenitors that recapitulate the architecture and functionality of specific organs or tissues. Organoids represent a paradigm shift in experimental modeling, offering a more physiologically relevant platform for studying organ development, disease pathology, and drug responses, compared to traditional two-dimensional (2D) cell culture systems. Regarding neurodegenerative diseases, both iPSCs and organoids offer a unique opportunity for studying patients’ cells, bypassing the obvious impossibility of using brain tissue as the primary human source. Moreover, despite animal models contributing to the comprehension of the molecular pathogenesis for this type of diseases and the fact that they are still broadly used, they often fail when pre-clinical results are translated into clinical trials. This failure could be partially explained by the fact that a substantial divergence in cell composition, organization, and cell–cell interactions in the brain regions exists between animals and humans, along with different transcriptional profiling across species. It is worth reporting that this is particularly true not only for neurons, but to a greater extent for non-neuronal cells, such as microglia, astrocytes, and oligodendrocytes [58,59]. Differences in the gene expression of endothelial cells and pericytes have been described in recent papers [60], as well as a substantial divergence in the immune system [61]. Nowadays, it is well-known that innate immune responses play a crucial role in neurodegenerative diseases. This phenomenon, indeed, is not only a consequence of inflammatory stimuli, such as the accumulation of protein aggregates in AD, PD, and HD, but it is also a concurrent cause of neurodegeneration itself, by fueling the inflammatory process, which becomes chronic in these pathologies [61,62,63].

Furthermore, it is particularly difficult to predict the clinical efficacy, toxicity, and side effects of drugs.

On the contrary, 3D organoids offer the possibility to better recapitulate human brain physiology and are attractive tools for the design of therapeutic interventions.

Finally, the brain size of humans is much bigger compared to rodents and motor neurons have got much longer axons, which in turns implies the complex transportation of cargoes, namely proteins, lipids, vesicles, and organelles, which allow communication between the cell body and long-distance synaptic terminals [63].

However, not all that glitters is gold and there are also some limitations associated with the use of organoids for the study of neurodegenerative diseases. First, there are no current standardized protocols to generate organoids, and organoid-to-organoid variation is the major factor responsible for poor reproducibility of experimental results.

In addition to this, the generation and maintenance of long-term culture is time consuming, expensive, and requires adequate and trained laboratory staff.

Another drawback is that organoids can show necrotic core cell death due to the insufficient diffusion of oxygen and nutrients, especially for organoids with a large spheroid diameter. Finally, some evident concerns about the use of organoids arise when studying late-onset diseases, such as AD and PD, whose symptoms appear to be evident with increasing age.

Due to the intrinsic characteristics of motor neuron cells, the projections of which can reach up to 1 m in length, cerebral 3D organoids are not appropriate models to investigate the mechanisms underlying motor neuron neurodegeneration, as well as synapse dysfunction at the neuromuscular junction level. However, some experiments aimed at exploring the functional role of the spatacsin gene, a form of HSP (SPG11) [64], in neurodevelopmental alterations were recently carried out [65]. Biallelic pathogenic variants in *SPG11* are responsible for the complicated form of SPG11, where most patients exhibit cognitive impairment and a thin corpus callosum, in addition to progressive spastic paraplegia. Mutations in this gene also cause a form of ALS (ALS5) and a form of Charcot–Marie–Tooth disease (CMT1a). Neurodegenerative phenotypes are linked to autophagy. Indeed, at a molecular level, spatacsin is involved in autophagic lysosomal reformation that is, in turn, crucial for lysosomal homeostasis during autophagy.

Pérez-Brangulí and colleagues, using 2D and 3D models, differentiating neural progenitor cells (NPCs), neurospheres, and cerebral organoids, from patient iPSCs carrying *SPG11* mutations, showed an increased level of asymmetric division in SPG11 NPCs in the germinal zone of cortical ventricles at the expense of symmetric division, shedding new light on the neurodevelopmental mechanism underlying this type of HSP. This impairment in self-renewal contributes to the premature generation of cortical neuroblasts and neurons and is dependent on GSK3 signaling. In summary, the authors showed that patient-derived organoids displayed proliferation defects, a reduced size, and premature neurogenesis. Interestingly, the administration of tideglusib, an FDA-approved GSK3 inhibitor, was able to rescue premature neurogenesis and, more importantly, increase the organoid size in SPG11 organoids [65]. This is another important piece of research showing how organoids can help in delineating specific pathogenetic mechanisms and how they can be used for drug discovery in terms of the patient’s specific genetic background.

In vitro models of neuromuscular junctions (NMJs) represent a crucial tool for the understanding of ALS [8,66] etiopathogenesis before the initial symptoms of the disease appear; nevertheless, as for HPS, 3D models to study trunk spinal neuromuscular neurodegeneration are difficult to conceive and to realize. However, some attempts have recently been made in this field. In 2021, Pereira and colleagues developed a spinal neuromuscular model represented by sensorimotor organoids containing physiologically functional NMJs from iPSC-derived ALS individuals and matched controls. These structures recapitulate physiological NMJ synapses, display motor and sensory neurons, astrocytes, and mesodermal derivatives, including vasculature, microglia, and skeletal muscle. Interestingly, NMJs derived from patients carrying pathogenic variants in all genes involved in the pathogenesis of ALS were impaired. In fact, a reduction in muscle contraction within ALS organoid cultures was reported, as well as hyperexcitability of neurons, which might contribute to NMJ degeneration in this type of neurodegenerative disorder [67].

Recently, another group generated a spinal neuromuscular model derived from C9orf72 ALS patients’ iPSCs, culturing them for 50 days, even though neuromuscular hallmarks start appearing earlier (20 days). Interestingly, this model could mimic the progressive peripheral neurodegeneration of ALS, as demonstrated by the impairment of neurons and astrocytes in the spinal cord, along with loss of Schwann cells. This in vitro model was an efficient/valuable model for screening potential drug candidates in a more physiologically relevant context, as demonstrated by the effect of the inhibitor of the unfolded protein response, GSK2606414. The administration of this drug, indeed, rescued the skeletal muscle defects and reduced dipeptide repeat protein aggregation and autophagy in a dose-dependent manner [68].

The same inhibitor was found to partially rescue early protein homeostasis and DNA damage-related pathogenesis in a long-term human cerebral organoid slice model, using iPSCs derived from patients with ALS overlapping with frontotemporal dementia that harbor the C9orf72 hexanucleotide repeat expansion mutation [69].

As described before, PD is a neurodegenerative disorder characterized by progressive dopaminergic neuron loss in the substantia nigra of the midbrain, usually presenting symptoms, such as tremors, impairment in motor coordination, muscle rigidity, and cognitive decline [2,70].

As for other neurodegenerative diseases, the causes that induce early neuron degeneration are still largely unknown and the development of 3D brain organoids would add to the available physiological models that help to understand PD pathogenesis and the identification of novel therapeutic options.

Human midbrain organoids (hMBOs) from a patient harboring the triplication of the *SNCA* gene, a rare Mendelian form of early-onset PD, accumulate pathological α-synuclein and this phenomenon is accompanied by a parallel loss of dopaminergic neurons and increased apoptosis [71].

Similarly, hMBOs with triplication of the *SNCA* gene generated by Muwanigwa and colleagues showed higher levels of intra and extracellular α-synuclein associated with a senescent-like phenotype in astrocytes, along with a progressive decline in dopaminergic neurons, suggesting that normal astrocytes are crucial for the maintenance of neuron fitness and homeostasis [72].

Midbrain organoids carrying the p.Gly2019Ser mutation in the *LRRK2* gene were used for studying sporadic PD, although this mutation is also associated with the autosomal dominant form of PD. Of interest, in this in vitro model, along with α-synuclein aggregates, the authors demonstrated the increased expression of the thioredoxin-interacting protein (TXNIP), a protein associated with lysosomal dysfunction in α-synuclein-overexpressed cultures [73]. Of note, *TXNIP* knockdown rescued α-synuclein oligomers in *LRRK2* p.Gly2019Ser knock-in 3D organoids, suggesting the importance of this gene in the development of *LRRK2*-associated Parkinson’s disease [74]. Moreover, a significant decrease in the expression of *NR2F1*, the negative regulation of *TXNIP* expression, was reported by the Schwamborn group in hMBOs derived from PD patients carrying the *LRRK2* p.Gly2019Ser mutation [75].

In iPSC-derived midbrain organoids deficient in terms of DJ1 activity, lysosomal processing of α-synuclein is impaired in astrocytes, confirming the key role played by this type of cell in neuroinflammation and neurodegeneration in PD. Indeed, the upregulation of inflammatory proteins, such as ANXA3, FGB, AMIGO2, and SERPINE1, as well as IL32 and IL18, was also observed. In parallel, alteration of the ubiquitin–proteasome system and autophagic flux were found in this system, in line with earlier reports [76].

Moreover, α-synuclein aggregation, an increase in the intrinsic neuronal firing frequency, abnormal function at mitochondrial and lysosomal levels, and final neurodegeneration, were found in hMBOs with mutations in *DNAJC6*. Mutations in this gene are present in early- and juvenile-onset PD (OMIM 608375) [77]. Of note, a decrease in *LMX1A* gene expression levels, during the early developmental period of hMBOs, rendered dopaminergic neurons more prone to degeneration [78].

Recently, to gain insights into the mechanisms whose disruption ends with dopaminergic loss, murine assembloids were employed because of their potential capacity to investigate complex cell–cell interactions and the circuit formation between cells from different brain regions in long-term cultures [79]. Assembloids, indeed, are two or more fused region-specific organoids that create a 3D structure, resembling more closely the intricate interactions between different types of cells in a specific organ.

In 2023, the first in vitro model of assembloids of the fused ventral midbrain and striatal cortex was developed [80]; then, in 2024, Variola’s group generated midbrain and striatal assembloids that allowed direct observation of the spatiotemporal patterns of the electrical activity occurring between the two organoids, without requiring neural tracing and calcium imaging within the 3D structure. They represent a promising tool for studying dopaminergic networks in PD patients and the effects of different therapeutic strategies [81].

In the last few years, the scientific literature regarding the use of organoids as a 3D model to study neurodegenerative diseases, particularly AD, has flourished.

In 2018, Gonzalez and colleagues reported that cerebral organoids obtained from iPSCs derived from familial AD patients and patients with Down syndrome can develop Tau pathology. These organoids, indeed, recapitulated some of the main features of AD, such as amyloid-β deposition that is highly reminiscent of amyloid plaques, the accumulation of p-tau, and increased apoptosis, especially in organoids containing the largest amount of Aβ and tau aggregates. These alterations are Alzheimer-specific, as they are absent in cerebral organoids from healthy individuals and from other neurodegenerative diseases [82].

A crucial role of *APOE4* in AD pathogenesis is highlighted by Zhao and colleagues; the authors obtained cerebral organoids from both cognitively unimpaired and impaired individuals carrying the *APOE* ε3/ε3 or ε4/ε4 genotype, demonstrating that *APOE* ε4/ε4 worsens tau pathology in both organoids from AD patients and healthy individuals, but larger amounts of ab and phospho-tau are found in AD patients. Of note, the conversion of *APOE4* into *APOE3* reduced the AD-related phenotype of organoids [83].

Cerebral organoids generated from patients with mutations in *PSEN1* and *PSEN2* exhibited AD-like features. In fact, they gradually accumulated Aβ, especially in organoids derived from a patient carrying the *APOE3/4* risk genotype, they displayed higher phosphorylation of tau, neuronal cell-type loss, increased cellular stress/apoptosis, and the induction of senescence. Finally, developmental and tissue patterning defects were described in AD iPSC-derived organoids [84].

To screen for blood–brain barrier permeable, FDA-approved drugs, Park and colleagues developed a drug assessment platform, using iPSC-derived cerebral organoids obtained from sporadic AD patients and healthy individuals. They created about 1300 organoids that were deeply characterized by analyzing the transcriptome, checking the levels of pathogenic proteins, such as Aβ and p-tau, measuring the intracellular calcium, and evaluating other hallmarks. Thanks to a systems biology approach, the researchers constructed and validated a model of the neuronal molecular regulatory network for AD that is a proof-of-principle screening of drugs that directly target AD-relevant cellular pathways and induce a reduction in Aβ and p-tau [85].

Cerebral organoids derived from iPSCs can be used to provide a 3D model of human brain development and disease. Organoids recapitulate key aspects of brain architecture and organization, allowing researchers to study complex interactions between different cell types, model disease progression, and screen for drugs that can modify disease phenotypes.

## 3. Current Challenges and Future Directions

Experimental cell models are indispensable tools in biomedical research, providing valuable insights into cellular mechanisms, disease pathogenesis, and therapeutic development. However, each model presents specific limitations and there are many areas for improvement (Table 1).

We are convinced that a patient’s specific cells can be a reliable model for disease investigation. However, their use is constrained by ethical considerations, limited availability, and variability between donors. Moreover, some limits lie within the very nature of the cell models. For instance, it is obvious that primary cell lines, cultured as two-dimensional monolayers, fail to replicate the complex structure and microenvironment of tissues and organs. These simple models do not account for the interactions between different cell types or the microenvironment effect. The possibility to collect different patient cells is also limited. This limit was overcome by the reprogramming of iPSCs that now allows us to differentiate these cells into different cell types. Moreover, iPSCs can be obtained from different patient’s cells. As we briefly discussed in the iPSC section, the process to generate these cells is expensive and it is characterized using multiple reprogramming factors. This means that often the pluripotent cell population generated is heterogeneous.

As reproducibility is of fundamental importance, this can be an important issue for its translation into clinical applications [86].

As reported in the iPSC section, we discussed how efforts have been made to compare reprogrammed cells with different methods to assess consistency. On top of that, this issue is being addressed by the creation of multiple biobanks, accompanied by detailed patient clinical records. This problem can also be partially solved by working with established iPSC lines. These cells can be modified by CRISPR/Cas editing technologies, in case specific variants have to be analyzed. Nevertheless, as we solve one side of the issue, on the other hand, this does not solve the complexity of the representation of individual (patient specific) genetic backgrounds. This could limit the understanding of disease mechanisms and eventually the development of personalized therapies.

Organoids represent unique models to study the molecular effects of genetic variants or the effect of drugs in terms of a complex 3D architecture. While these models represent sophisticated systems, many limitations are present. In fact, although the cell types in organoids largely represent human cell types according to their expression profiles, the related specification programs are compromised. The complexity in gene expression levels and in the networks of gene interactions, which characterize natural development, is far from being recapitulated by cells derived from organoids, an important aspect when dealing with brain organoids. Other common issues in organoid modeling are represented by size issues; the lack of vascularization, which limits nutrient delivery; metabolite elimination (especially in intestinal crypt organoids); and cell signaling [87].

Each model has its limitations and organoids are no exception. As for other models, the method of culturing or reprogramming human cells must be consistent and reproducible, ideally using isogenic controls or multiple biological and technical replicates. The use of biobanks with characterized models and clinical records is also a strategy to mitigate differences, with the understanding that models are an approximation.

Future directions in the field of experimental cell modeling include the adoption of standard strategies for the collection, reprogramming, and generation of 3D cell models It is auspicial that the use of a biobank that is accessible to each researcher, with clinical descriptions, would be a valuable approach that could be expanded for different conditions and could become the basis for future modeling strategies. This must be taken into consideration and interpreted with caution when being translated in regard to in vivo models and human physiology.

## 4. Discussion

Substantial investments have been made in researching neurodegenerative diseases; one of main limits in studying neurodegenerative disease has always been finding a proper cell/tissue source to employ to pursue this aim. For a long time, in fact, brain-based findings in humans have been obtained through post-mortem analysis, or in vivo imaging approaches, due to the problems related to tissue accessibility. Employing alternative cells to model these diseases is a valid strategy; as previously described, each strategy has its own limitations and advantages. Collectively, primary patient cell lines (mainly derived from fibroblasts), iPSCs, and organoids, have overcome many problems related to tissue accessibility, by providing an alternative source of cells. By using these alternative sources, ethical issues related to the use of ESCs (embryonic stem cells) have also been addressed. Moreover, using patient-derived cells to model disease and implement therapeutical strategies, further increases the compatibility of cell transplant-based therapies and the reliability of such models.

Despite the collective efforts by the scientific community to create different kinds of models to represent neurodegenerative diseases, some issues remain unsolved to date. The main limitations often result in a lack of phenotype–genotype correlations. This lack of correlation could be caused by various reasons; in primary cell models, the chosen tissue seems to be key to recapitulating the disease features. On the other hand, in iPSCs and organoids there is the loss of the epigenetic signature and, particularly, the age-related epigenetic signature could be an important factor in disease-modeling approaches. Another topic that needs to be addressed is the standardization of protocols, which would facilitate all the previously mentioned applications, making them more detailed, applicable, and comparable. All these issues have been highlighted and have started to be addressed by different research groups.

Despite these limitations, experimental models represent a fundamental tool in neuroscience research. Experimental models from primary patient cell lines to sophisticated organoid models can be employed to shed light on pathogenetic conditions and, eventually, to identify therapeutic interventions for this group of currently incurable diseases.

## 5. Conclusions

The experimental cell models described in this review have unique advantages and disadvantages, but they complement each other and offer unique opportunities for studying different aspects of neurodegenerative diseases, from the molecular mechanisms to more complex phenotype manifestations. Integrating the findings from these diverse models enhances our understanding of disease pathogenesis and accelerates the development of effective therapies for these devastating disorders.

## Figures and Tables

**Table 1:** Schematic representation of main advantages and disadvantages of the described models.

Cell Model	Advantage	Disadvantage
**Fibroblasts**	Easy to harvest	Lacks dimensional complexity
	Standardized protocols	Lacks neuron tissue specific features key for deep characterization
	Expression profile similar to neurons	
	Minimally invasive to collect	
	Important for basic characterization	
	Useful platform for drug screening	
**iPSCs**	Derived from somatic cells	Lacks dimensional complexity
	Can be source of cell therapy and derived directly from the recipient	Poor standardization of protocols
	Important for deep characterization thanks to tissue-specific features	Lacks epigenetic age and correlation of phenotypes related to it
	Can generate neural cells without invasive surgical approaches	Diseases with complex genetic features are hard to address
	Useful platform for drug screening	Expensive maintenance
	Useful for early phenotype correlation	
**Organoids**	Adds dimensional complexity to disease modeling	Core of necrotic cells
	Can be more reliable than animal models in drug testing and in recapitulating disease features	Poor standardization protocols
	Can recapitulate complex interactions and brain physiology	Lacks epigenetic age and correlation between phenotypes related to it
		Hard and expensive maintenance
